# Canadian Pediatric Weight Management Registry (CANPWR): baseline descriptive statistics and comparison to Canadian norms

**DOI:** 10.1186/s40608-015-0060-6

**Published:** 2015-08-13

**Authors:** Mark S. Tremblay, Min Feng, Didier Garriguet, Geoff D. C. Ball, Annick Buchholz, Jean-Pierre Chanoine, Marie Lambert, Katherine M. Morrison

**Affiliations:** Healthy Active Living and Obesity Research Group, Children’s Hospital of Eastern Ontario Research Institute and Department of Pediatrics, University of Ottawa, Ottawa, Canada; Population Health Research Institute, McMaster University, Hamilton, Canada; Health Analysis Division, Statistics Canada, Ottawa, Canada; Stollery Children’s Hospital, Alberta Health Services and Department of Pediatrics, University of Alberta, Edmonton, Canada; Centre for Healthy Active Living, Children’s Hospital of Eastern Ontario, Ottawa, Canada; British Columbia Children’s Hospital, Department of Pediatrics, University of British Columbia, Vancouver, Canada; CHU Sainte Justine’s Children’s Hospital, Montreal, Canada; McMaster Children’s Hospital, Department of Pediatrics, and Population Health Research Institute, McMaster University, Hamilton, Canada; CHEO Research Institute, 401 Smyth Road, Ottawa, K1H 8L1 Canada

**Keywords:** Obesity, Overweight, Child, Youth, Health, Risk factor

## Abstract

**Background:**

A pilot study was conducted to assess the feasibility of establishing a multi-site CANadian Pediatric Weight management Registry (CANPWR) containing individual, family and weight management program information.

**Methods:**

Standardized baseline data were collected to characterize CANPWR participants (n = 310) in comparison to a sample of age-matched Canadian children measured in the nationally representative Canadian Health Measures Survey (CHMS; n = 3,788). This study compared demographic, anthropometric, cardiometabolic and lifestyle characteristics of participants (aged 6–17 years) in the CANPWR pilot study with those from the CHMS.

**Results:**

Compared to CHMS respondents, CANPWR participants had higher BMI z-score, waist circumference, blood pressure, total cholesterol, LDL cholesterol, triglycerides and fasting glucose, and lower HDL cholesterol. They reported lower sugared drink consumption, were more likely to be non-white and had parents with lower education.

**Conclusions:**

The CANPWR cohort represents a group that has biological and behavioral profiles that place them at increased health risk and who differ significantly from typical Canadians of the same age.

## Background

The current prevalence of childhood obesity in Canada is 11.7% [1]. A number of tertiary care centres in Canada offer pediatric weight management programs, although programs are not linked or harmonized and their efficacy and effectiveness is largely unknown [2, 3]. To address these limitations, the CANadian Pediatric Weight management Registry (CANPWR) [2] was created to:

- Document changes in anthropometric, lifestyle, behavioral, and obesity-related co-morbidities in children enrolled in Canadian pediatric weight management programs over a three-year period;
- Characterize the individual-, family-, and program-level determinants of change in anthropometric and obesity-related co-morbidities;
- Examine the individual-level, family-level, and program-level determinants of program attrition.

CANPWR is a prospective cohort, multi-centre study that includes children 2–17 years old with body mass index ≥85th percentile who are enrolled in one of eight Canadian pediatric weight management centres. CANPWR aims to recruit 1,600 study participants over a three-year period with data collection planned at baseline and 6-, 12-, 24-, and 36-months follow-up. The study outcomes of interest include BMI z-score, change in BMI z-score over time, anthropometric, cardiometabolic, lifestyle, and psychosocial variables as well as potential determinants of change and program attrition at the individual-, family-, and program-level. Further details on the CANPWR study and protocol are available elsewhere [2].

Using baseline data from five sites who participated in this CANPWR pilot study, we aimed to characterize CANPWR participants at enrolment into a weight management program in comparison to a sample of age-matched Canadian children measured in the nationally representative Canadian Health Measures Survey (CHMS) [4, 5].

## Methods

This study included participants enrolled in the CANPWR pilot study. Research Ethics Board approval was obtained for the CANPWR study from all participating sites [2]. Written informed consent was provided for all participants by their parents or guardian and written participant assent was also obtained. Participants were from one of five Canadian pediatric weight management centres (Montreal, Ottawa, Hamilton, Edmonton, Vancouver) and the baseline measures used in this analysis were gathered between January 2011 and December 2012 at the time they entered the weight management program and before initiating obesity management. Inclusion criteria for enrolment in the weight management programs differed among sites; all sites required a BMI cut-off (one was >85^th^ centile, three were >95^th^ centile, and one was >99^th^ centile or 95^th^ centile with a comorbidity). Participants also needed to have English or French language comprehension, geographic availability to attend the program appointments, be 6–17 years of age (for this study) and have parental participation.

Harmonized measures from the CHMS and CANPWR included demographic information (age, sex, cultural background, country of birth, household income, household highest education) and measures of anthropometry (height, weight, waist circumference), cardiometabolic disease biomarkers (systolic and diastolic blood pressure, total cholesterol, triglycerides, LDL cholesterol, HDL cholesterol, fasting glucose, glycosylated hemoglobin (HbA1c%), alanine aminotransferase (ALT), aspartate transferase (AST)) and lifestyle behaviours (reported food frequency consumption, physical activity, screen time, sleep). Questionnaires were completed by parents and child together (CANPWR and CHMS for children <12 years of age) or by the child alone (CHMS ages ≥12 years of age). The questions for reported variables used in this manuscript are provided in Table 1. All CANPWR blood samples were collected in the fasted state. The CHMS participants were split between morning (fasted) and afternoon (not fasted): the results for triglycerides, LDL cholesterol and glucose were tested on the fasted sample only while the other biomarkers were based on the total sample (fasted and non-fasted). Further details of the CHMS [4, 5] and CANPWR measures are available elsewhere [2]. Data were captured in a centralized database after ethical clearance was obtained and data transfer agreements and contracts were in place with all participating sites. The pilot study included data from 310 CANPWR participants aged 6–17 years.

**Table 1:** Questionnaire questions used for reported variables

Item	Question	Response choices
Cultural background	People living in Canada come from many different cultural and racial backgrounds. Is the child:	White, Chinese, Black, Filipino, Latin American, Canadian Frist Nation, Southeast Asian, Arab, West Asian, Japanese Korean, Other
Country of Birth	In what country was the child born?	Canada, China, France, Germany, Greece, Guyana, Hong Kong, Hungary, India, Italy, Jamaica, Netherlands/Holland, Philippines, Poland, Portugal, United Kingdom, United States, Vietnam, Sri Lanka, Other
Household Income	Please indicate which category best represents the total annual household income from all sources (CAN currency)	Less than $49,000, $50,000-79,999, $80,000-99,999, $100,000 and above, not available
Education	What is the highest level of education attended by this child’s female and male primary caregiver?	No high school, Some high school, High School diploma, University/college, Post graduate, not available
Sleep – difficulty falling asleep or staying asleep	How often does this child have trouble going to sleep or staying asleep?	Never, Rarely, Sometimes, Most of the time, All of the time, Don’t Know
Sleep – difficulty to stay awake	How often does this child find it difficult to stay awake during normal waking hours?	Never, Rarely, Sometimes, Most of the time, All of the time, Don’t Know
Sleep – mean hours per day	How many hours does this child usually spend sleeping in a 24 h period, excluding time spent resting?	Reported in ‘x’ number of hours
Physical activity – 60 min daily	Over a typical or usual week, on how many days is the child physically active for a total of at least 60 min per day?	None, 1 day, 2 to 3 days, 4 days or more, Don’t know
Screen time	On average, about how many hours a day does the child spend on total screen time? (T.V, Computer, Video games, smartphones, social media etc.)	Doesn’t use a computer or play video games, <1 h/day, 1 to 2 h/day, 3 to 4 h/day, 5 to 6 h/day, 7 or more hours/day
Food Intake – Overarching question:		
How often does your child usually eat the following foods (both meals and snacks, at home and away from home),		
Food intake – Milk consumption	Milk (3%, 2%, 1%, Skim, Flavoured, Rice, Soya)	Never, less than once/month, 1 – 3/month, 1/week, 2–4/week, 5-6/week, 1/day, 2-3/day, 4-5/day, >6/day
Food intake – Dairy consumption	Dairy (Cottage Cheese, Yogurt, Ice cream, frozen yogurt, hard cheese	As above
Food intake – fruit and vegetable consumption	Fruit, lettuce or green leafy salad, spinach, mustard greens or collards, Other types of vegetables	As above
Food intake – sugared drink consumption	Regular soft drinks, Sports Drinks, Energy drinks, Fruit juice, fruit flavoured drinks	As above
Food intake – water consumption	Beverages, water	As above

NOTE: For nutritional intake a typical serving size is provided, each item is separate and the total are added together

The comprehensive set of baseline characteristics, behaviours, and biomarkers of CANPWR participants was compared to same-aged children and youth (n = 3,788) measured in the nationally representative CHMS [4, 5] conducted by Statistics Canada over the same period. The CHMS sample was selected based on age, sex and geographic region. Sample weights were calculated and used in the analysis (more details are available in the CHMS Data User Guide: Cycle 2 November 2012; available on request at: http://www23.statcan.gc.ca/imdb-bmdi/document/5071_D4_T9_V1-eng.htm). Research Ethics Board approval for the CHMS was obtained from Health Canada and included procedures for obtaining written assent from participants and consent from parents or guardians [6]. Questions and measures were harmonized between the CHMS and CANPWR samples. CANPWR sample means, for ages 6–11 years and 12–17 years, were compared with CHMS 95% confidence intervals using Student’s *t*-test for continuous variables; CANPWR percentages were compared with CHMS percentages using Chi-square test for categorical variables. Statistical significance was set at *p* < 0.05. A specific power analysis was not performed for each measure, however with the majority of variables showing significant group differences, the sample was deemed sufficient to describe differences between the CHMS and CANPWR samples.

## Results

Socio-demographic, anthropometric, behavioral and physiological phenotype characteristics for CANPWR and CHMS samples are presented and compared in Table 2. The CANPWR sample was less likely to be male (12–17 year-olds) or White Caucasian. CANPWR participants were less likely to come from a household with ≥ $100,000 annual income or caregivers with a university education. As expected, the CANPWR sample was heavier and had a higher body mass index z-score (BMIz) and waist circumference than the CHMS sample. Consistent with these anthropometric differences, the CANPWR participants had higher resting blood pressure, total cholesterol, LDL cholesterol, triglycerides and fasting glucose, and lower HDL cholesterol, but had no difference in HbA1c or alanine aminotransferase (ALT). The CANPWR sample had lower sleep quantity (among 6–11 year-olds), but reported less difficulty going to sleep or staying awake. The proportion of the CANPWR sample adhering to screen time guidelines [7] was much lower than in the CHMS. There were no statistically significant differences in the proportion of participants that reported accumulating ≥60 min of physical activity at least four days per week. The CANPWR sample reported more daily servings of vegetables and fruit, as well as milk, and fewer servings of sugar-sweetened beverages.

**Table 2:** Comparison of CANPWR sample versus CHMS for children aged 6–11 years and youth aged 12–17 years

Measure	CANPWR		CHMS	
Age range (years) (sample size)	6-11 (n = 171)	12-17 (n = 139)	6-11 (n = 2138)	12-17 (n = 1650)
Categorical variable analyses (Chi-Square comparisons)				
Male (%)	52.0	**42.4**	51.3	53.0
Cultural background White (%)	**65.5**	**71.9**	77.1	80.0
Country of birth Canada (%)	88.3	85.6	90.3	90.0
Household income ≥ $100,000 (%)	**24.6**	**28.1**	33.0	36.7
Household highest education University/College (%)	**62.0**	**58.3**	86.2	85.9
Never have difficulty to go to sleep (%)	**44.4**	**43.9**	37.1	22.0
Never have difficulty to stay awake (%)	**63.2**	**58.3**	71.0	37.0
Physical activity at least 60 min daily ≥4 days/week (%)	38.0	24.5	33.8	19.2
Total screen time ≤2 h/day (%)	**32.8**	**13.0**	65.8	44.7
Continuous variable analyses (Student’s *t*-test comparisons), mean (SD)				
Age (years)	**9.5 (1.4)**	**14.1 (1.8)**	8.6 (1.7)	14.5 (1.7)
Height (cm)	**146.2 (11.2)**	165.2 (10.7)	135.0 (12.0)	165.0 (10.0)
Weight (kg)	**62.6 (16.6)**	**99.5 (24.8)**	33.4 (10.7)	60.6 (16.9)
BMI z-score	**3.64 (1.08)**	**3.43 (1.30)**	0.55 (1.25)	0.43 (1.27)
Waist circumference (cm)	**88.2 (13.9)**	**105.1 (18.1)**	60.8 (9.8)	73.9 (12.4)
Systolic blood pressure (mmHg)	**107 (11)**	**116 (12)**	94 (8)	97 (8)
Diastolic blood pressure (mmHg)	**66 (7)**	**70 (8)**	61 (8)	62 (7)
Total cholesterol (mmol/L)	**4.39 (0.75)**	**4.27 (0.79)**	4.23 (0.69)	4.06 (0.76)
Fasting LDL cholesterol (mmol/L)	**2.59 (0.62)**	**2.55 (0.63)**	2.36 (0.67)	2.28 (0.73)
HDL cholesterol (mmol/L)	**1.23 (0.32)**	**1.13 (0.42)**	1.42 (0.31)	1.31 (0.30)
Fasting triglycerides (mmol/L)	**1.35 (0.89)**	**1.48 (0.71)**	0.82 (0.39)	0.97 (0.49)
Fasting glucose (mmol/L)	**4.89 (0.33)**	**5.05 (0.97)**	4.60 (0.87)	4.70 (0.65)
HbA1c (%)	5.45 (0.32)	5.49 (0.75)	5.46 (0.36)	5.43 (0.35)
Alanine aminotransferase (ALT)(IU/L)	27.0 (19.6)	26.9 (18.4)	27.8 (19.4)	27.3 (10.4)
Aspartate aminotransferase (AST)(IU/L)	**27.4 (10.6)**	**24.2 (10.6)**	33.8 (16.9)	27.0 (8.4)
Milk consumption (times/day)	**1.9(1.3)**	**2.2(2.2)**	1.7(1.6)	1.4(1.6)
Total dairy consumption (times/day)	2.7 (1.6)	**3.1 (2.5)**	2.6 (2.0)	2.0 (2.0)
Total fruit and vegetable consumption (times/day)	**3.8 (2.5)**	**4.0 (2.5)**	3.2 (1.8)	3.0 (2.3)
Water consumption (times/day)	**3.9 (2.7)**	3.3 (2.5)	3.4 (2.4)	3.3 (2.4)
Daily sugared drink consumption (times/day)	**1.0 (1.1)**	**1.0 (1.0)**	1.5 (1.1)	1.6 (1.3)
Mean sleeping hours per day	**9.4 (1.1)**	8.3 (1.2)	9.6 (1.0)	8.4 (1.2)

Note: Bold text indicates statistically significant difference (*p* < 0.05) from same age category from CHMS

## Discussion

The purpose of the weight management programs participating in CANPWR is to both reduce risk for obese children already presenting with adverse health risk profiles as well as to mitigate the development of adverse health risk profiles among obese children without evident comorbidities. The CANPWR pilot contains a sample of children and youth who have characteristics and phenotypes that are distinctly different than their age-matched nationally representative Canadian peers. Socio-economic status was generally lower in the CANPWR sample, consistent with gradients observed in other developed countries [8]. Physiological biomarkers were noticeably different between groups with the CANPWR sample having biologically meaningful unfavorable cardiometabolic disease risk profiles, consistent with those predictive of future adverse health outcomes [9, 10]. This finding supports aggressive management and treatment programs for children and youth with obesity who present with adverse health risk profiles or existing co-morbidities. Furthermore, this observation supports the need for intensive efforts targeted towards childhood obesity prevention.

Some reported healthy behaviour characteristics were better in the CANPWR sample, while others were worse compared to the CHMS sample. This is an interesting finding in light of the fact that the measures were taken *before* participants started health services for obesity management. Particularly encouraging is the reporting of frequent consumption of vegetables and fruit as well as milk. The finding of significantly lower sugared drink consumption was unexpected and discordant with other studies [11–13]. It is possible that prior to enrolment in the weight management program, CANPWR participants had been counseled to reduce sugared drink consumption. It is also worth noting that the reporting of behavioral measures is susceptible to social desirability bias [14, 15] and objective measures should be used to verify these findings with future enrollees. Obese children may be more susceptible to such reporting biases. Alternatively, it is possible these youth are accurately reporting their behaviors, and the reason they are seeking out tertiary care treatment is that efforts to implement healthier nutritional intake have not worked for them. The CANPWR participants were less likely to meet established screen time guidelines [7], which is relevant since excessive screen time, especially in the form of television viewing, has been linked to increased risk of obesity [16, 17] and increased caloric intake [18]. This may be an important modifiable lifestyle behavior to target. Reported physical activity was comparable between samples highlighting the importance of examining both physical activity and sedentary behaviours when assessing potential intervention strategies [19]. It is also possible that obese children are more likely to over-report their physical activity [20] which may mask true differences.

## Conclusions

The CANPWR participants were socioeconomically, behaviorally and physiologically different than typical age-matched Canadian children and youth. The CANPWR cohort represents a group that has biological and behavioral profiles that place them at increased health risk compared to typical Canadians of the same age. These observations support the value of establishing a multi-site pediatric weight management registry of children and youth with obesity, not only to compare weight management program characteristics and outcomes, but to create a platform for future research into the biological, psychological and behavioral trajectory of Canadian children and youth who are exposed to a variety of obesity management programs across the country.
